# Supplementary material for: Gestational overweight decreased risk of antepartum hemorrhage in pregnant women with complete placenta previa: a retrospective study
Source: PeerJ. 2025 Feb 25;13:e19091. doi: 10.7717/peerj.19091 (PMC11869886; doi:10.7717/peerj.19091)
Supplement: Supplemental Information 2 [file peerj-13-19091-s002.doc]

Supplementary Table 1. Logistic regression model to assess the APH and potential variables.

| Covariates | Crude | | |  | Adjusted* | | |
| --- | --- | --- | --- | --- | --- | --- | --- |
|  | OR | 95% CI | *P-*values |  | OR | 95% CI | *P-*values |
| With complication |  |  |  |  |  |  |  |
| Weight (kg) | 0.946 | 0.913-0.991 | 0.016 |  | 0.961 | 0.901-0.997 | 0.042 |
| BMI (kg/m^2^) | 0.826 | 0.713-0.946 | 0.012 |  | 0.865 | 0.718-0.978 | 0.031 |
| Overweight (n) | 0.574 | 0.216-0.848 | 0.019 |  | 0.563 | 0.215-1.125 | 0.095 |
| Need for blood transfusion (%) | 2.158 | 1.301-4.768 | 0.009 |  | 1.145 | 0.608-3.912 | 0.396 |
| Length of hospital stay (days) | 1.016 | 1.017-1.184 | 0.006 |  | 1.005 | 0.990-1.193 | 0.297 |
| Weight (g) | 0.979 | 0.991-0.999 | 0.001 |  | 0.991 | 0.989-1.001 | 0.058 |
| Without complication |  |  |  |  |  |  |  |
| Weight (kg) | 0.911 | 0.863-0.981 | 0.016 |  | 0.915 | 0.801-0.996 | 0.038 |
| BMI (kg/m^2^) | 0.802 | 0.711-0.924 | 0.015 |  | 0.814 | 0.723-0.978 | 0.016 |
| Overweight (n) | 0.413 | 0.124-0.861 | 0.029 |  | 0.498 | 0.205-1.127 | 0.099 |
| Need for blood transfusion (%) | 2.628 | 1.401-4.964 | 0.009 |  | 1.545 | 0.608-3.902 | 0.436 |
| Length of hospital stay (days) | 1.168 | 1.012-1.314 | 0.013 |  | 1.096 | 0.989-1.280 | 0.325 |
| Weight (g) | 0.999 | 0.993-0.999 | 0.015 |  | 1.001 | 0.993-1.002 | 0.086 |

*Adjust for gestation week, primiparous, prior cesarean delivery, and prior abortion.

Supplementary Table 2. The relationship between maternal BMI categories and specific outcomes (e.g., gestational age or blood transfusion rates).

|  | gestational age (weeks) | blood transfusion rates (%) |
| --- | --- | --- |
| Normal weight 78 (43.1) | 34.33±1.85 | 50 (64.1) |
| Overweight 89 (49.2) | 37.12±0.77 | 41 (46.1) |
| Obese 14 (7.7) | 39.25±0.91 | 7 (50.0) |
| *P*-values | 0.001 | 0.064 |
| *F/χ^2^* | 128.714 | 0.551 |
