# Supplementary material for: Gestational overweight decreased risk of antepartum hemorrhage in pregnant women with complete placenta previa: a retrospective study
Source: PeerJ. 2025 Feb 25;13:e19091. doi: 10.7717/peerj.19091 (PMC11869886; doi:10.7717/peerj.19091)
Supplement: Supplemental Information 3 [file peerj-13-19091-s003.doc]

**For providing detailed, valid reasons for the new author’s contributions.**

Dear Editors and Reviewers,

We thank the reviewers for their generous comments on the manuscript and have edited the manuscript to address their concerns.

We believe that the manuscript is now suitable for publication in PeerJ.

Shaoxin Ye, the new author, have made sufficient authorship contribution to the revision and all authors agree to add Shaoxin Ye to the list of authors.

Her main contributions were:

1) She explained the responses to critical questions asked by reviewers 2 and 3 concerning clinical practice, obstetric management, and the high proportion of PAS.
2) She led the effort to comb through the database and reanalyze the data, and the completed the supplement table 1 and table 2.

Given her contributions in the revision process, on behalf of all authors, I kindly ask the editors and reviewers to agree to list Shaoxin Ye as an author. All authors agree to the changes, and all our current coauthors have agreed with the change by responding to the change confirmation email.

Sincerely,

Dazhi Fan

And, see below for details of Shaoxin Ye’s contributions.

**Reviewer 2 (Anonymous)**

*Suggestions:
1/Add a sensitivity analysis or stratified analysis to evaluate potential confounders, such as the influence of gestational diabetes or hypertensive disorders.*

**Response:** Thank you very much for this suggestion. We further performed a subgroup analysis according to the presence or absence of complications, and the results were not materially altered. The supplement table 1 showed the details data.

2/Provide more discussion on the clinical relevance of the findings, especially for obstetric management.

**Response:** Thank you very much for this suggestion. We added some sentences in the seventh part of discussion to discuss the clinical relevance of the findings.

**In line 218:** “The findings from this research will serve as a crucial reference for healthcare providers in establishing individualized weight gain guidelines tailored to optimize maternal and fetal health for pregnant women diagnosed with complete placenta previa. By understanding the specific needs and risks associated with complete placenta previa, medical professionals can provide more targeted and effective prenatal care. This approach not only aims to improve immediate pregnancy outcomes but also supports long-term health benefits for both mother and child, emphasizing the importance of personalized medicine in obstetric practice. Additionally, these insights can inform the development of clinical protocols and patient education materials, ultimately contributing to better health management and decision-making during pregnancy.”

*The results are clearly organized, but further exploration of the relationship between maternal BMI categories and specific outcomes (e.g., gestational age or blood transfusion rates) could be beneficial.*

**Response:** Thank you very much for this suggestion. We further performed analysis BMI categories and the specific outcomes, including gestational and blood transfusion rates. The supplement table 2 showed the details data.

**Reviewer 3 (Eva Pajkrt)**

- Please provide guidance for clinical practice since the mean BMI difference is small (1.7 BMI points), APH compared to non-APH.

**Response:** Thank you very much for your reminder. We added some sentences in the seventh part of discussion to provide guideance for clinical practice in the revised manuscript.

**In line 218:** “The findings from this research will serve as a crucial reference for healthcare providers in establishing individualized weight gain guidelines tailored to optimize maternal and fetal health for pregnant women diagnosed with complete placenta previa. By understanding the specific needs and risks associated with complete placenta previa, medical professionals can provide more targeted and effective prenatal care. This approach not only aims to improve immediate pregnancy outcomes but also supports long-term health benefits for both mother and child, emphasizing the importance of personalized medicine in obstetric practice. Additionally, these insights can inform the development of clinical protocols and patient education materials, ultimately contributing to better health management and decision-making during pregnancy.”

- Please provide insight into the high proportion of PAS (especially when taking into account only 18 women had a prior cesarean section).

**Response:** Thank you very much for your reminder. We added some sentences in the sixth part of discussion to provide insight into the high proportion of PAS.

**In line 211:** “It should be noted that the proportion of PAS is relatively high, reaching 8.3% in this study, much higher than 0.22% in Mainland China (Fan et al., 2017a) and 0.17% in the worldwide (Jauniaux et al., 2019b). The two main risk factors for PAS are prior cesarean section and placenta previa. Meanwhile, advancing maternal age, in vitro fertilization, multiparity, smoking and a short interval between a previous cesarean delivery and the subsequent pregnancy can also increase the risk of PAS (Wang et al., 2018; Liu et al., 2021; Premkumar et al., 2025). The high incidence of placenta accreta spectrum disorders in a dataset where only 18 (9.3%) of cases had previous cesarean deliveries further underscores the significant role that placenta previa plays in the development of PAS.”

Supplementary Table 1. Logistic regression model to assess the APH and potential variables.

| Covariates | Crude | | |  | Adjusted* | | |
| --- | --- | --- | --- | --- | --- | --- | --- |
|  | OR | 95% CI | *P-*values |  | OR | 95% CI | *P-*values |
| With complication |  |  |  |  |  |  |  |
| Weight (kg) | 0.946 | 0.913-0.991 | 0.016 |  | 0.961 | 0.901-0.997 | 0.042 |
| BMI (kg/m^2^) | 0.826 | 0.713-0.946 | 0.012 |  | 0.865 | 0.718-0.978 | 0.031 |
| Overweight (n) | 0.574 | 0.216-0.848 | 0.019 |  | 0.563 | 0.215-1.125 | 0.095 |
| Need for blood transfusion (%) | 2.158 | 1.301-4.768 | 0.009 |  | 1.145 | 0.608-3.912 | 0.396 |
| Length of hospital stay (days) | 1.016 | 1.017-1.184 | 0.006 |  | 1.005 | 0.990-1.193 | 0.297 |
| Weight (g) | 0.979 | 0.991-0.999 | 0.001 |  | 0.991 | 0.989-1.001 | 0.058 |
| Without complication |  |  |  |  |  |  |  |
| Weight (kg) | 0.911 | 0.863-0.981 | 0.016 |  | 0.915 | 0.801-0.996 | 0.038 |
| BMI (kg/m^2^) | 0.802 | 0.711-0.924 | 0.015 |  | 0.814 | 0.723-0.978 | 0.016 |
| Overweight (n) | 0.413 | 0.124-0.861 | 0.029 |  | 0.498 | 0.205-1.127 | 0.099 |
| Need for blood transfusion (%) | 2.628 | 1.401-4.964 | 0.009 |  | 1.545 | 0.608-3.902 | 0.436 |
| Length of hospital stay (days) | 1.168 | 1.012-1.314 | 0.013 |  | 1.096 | 0.989-1.280 | 0.325 |
| Weight (g) | 0.999 | 0.993-0.999 | 0.015 |  | 1.001 | 0.993-1.002 | 0.086 |

*Adjust for gestation week, primiparous, prior cesarean delivery, and prior abortion.

Supplementary Table 2. The relationship between maternal BMI categories and specific outcomes (e.g., gestational age or blood transfusion rates).

|  | gestational age (weeks) | blood transfusion rates (%) |
| --- | --- | --- |
| Normal weight 78 (43.1) | 34.33±1.85 | 50 (64.1) |
| Overweight 89 (49.2) | 37.12±0.77 | 41 (46.1) |
| Obese 14 (7.7) | 39.25±0.91 | 7 (50.0) |
| *P*-values | 0.001 | 0.064 |
| *F/χ^2^* | 128.714 | 0.551 |
